# Ursodeoxycholic Acid Confers Neuroprotection in Rats With Acute Cervical SCI and Attenuates NLRP3/Caspase‐1/GSDMD Pyroptotic Signaling

**DOI:** 10.1002/jbt.71157

**Published:** 2026-10-28

**Authors:** Honghu Song, Xiaomin Hu, Dan Liu, Linjie Wang, Li Zhu, Wenchun Wang, Rongnan Hu, Jiayan Zhong, Jiancheng Liu, Rizhao Pang, Anren Zhang

**Affiliations:** ^1^ School of Health Preservation and Rehabilitation Chengdu University of Traditional Chinese Medicine Chengdu China; ^2^ Department of Rehabilitation Medicine General Hospital of Western Theater Command Chengdu China; ^3^ College of Medicine Southwest Jiaotong University Chengdu China; ^4^ Department of Rehabilitation Medicine Shanghai Fourth People's Hospital Affiliated to Tongji University Shanghai China

**Keywords:** NLRP3/Caspase‐1/GSDMD pathway, pyroptosis, spinal cord injury, ursodeoxycholic acid

## Abstract

Spinal cord injury (SCI) leads to significant and enduring functional limitations for patients. Secondary injury is a pivotal determinant of functional recovery after SCI, with pyroptosis driven by the NLRP3 inflammasome and its downstream inflammatory cascade representing a crucial contributor to this process. Ursodeoxycholic acid (UDCA), an endogenous secondary bile acid, displays neuroprotective activity through antioxidant and anti‐inflammatory actions and may suppress pyroptosis. This study aimed to explore the neuroprotective effects of UDCA against SCI and its underlying mechanisms. BV2 microglia were treated with LPS and UDCA, either individually or concurrently. Cell viability and NLRP3 inflammasome protein expression were evaluated by CCK‐8 assay and immunofluorescence, respectively. Additionally, following establishment of a rat model of C5 cervical SCI, UDCA was administered by oral gavage for 7 days. Functional recovery, histopathological damage, key protein expression in the NLRP3/Caspase‐1/GSDMD pyroptosis pathway, and downstream inflammation were analyzed using various techniques, including behavioral evaluation (Cylinder Rearing test), HE staining, Western blot, immunofluorescence, and ELISA. Results showed that appropriate concentrations of UDCA improved BV2 cell viability and inhibited LPS‐stimulated NLRP3 upregulation. Oral gavage of UDCA was associated with better forelimb motor function and attenuated spinal histopathological injury in rats with acute C5 cervical SCI. Furthermore, this treatment correlated with altered abundance of key components within the SCI‑activated NLRP3/Caspase‑1/GSDMD pathway, lower Iba‑1 expression, and diminished serum concentrations of IL‑18, IL‑1β, and TNF‑α, suggestive of attenuated neural pyroptosis and dampening of excessive postinjury inflammation. Collectively, UDCA mitigates secondary injury and improves forelimb function in an acute C5 cervical SCI model, alongside attenuation of NLRP3/Caspase‐1/GSDMD‐mediated pyroptosis and subsequent inflammation. Targeting pyroptosis, UDCA holds promise as a novel therapeutic candidate for SCI.

## Graphical abstract

Ursodeoxycholic Acid could inhibit activation of the NLRP3/Caspase‐1/GSDMD pyroptosis pathway and reduce the release of downstream inflammatory cytokines IL‐18 and IL‐1β in rats with acute C5 cervical SCI. This modulation is associated with attenuated secondary injury and improved functional outcomes.

**Abbreviations**
Caspase‐1: cysteinyl aspartate specific proteinase 1
DMSO: dimethyl sulfoxide
ELISA: enzyme linked immunosorbent assay
GSDMD: gasdermin D
HE: hematoxylin‐eosin
Iba‐1: ionized calcium‐binding adapter molecule 1
IL‐18: interleukin‐18
IL‐1β: interleukin‐1β
LPS: lipopolysaccharides
NLRP3: NOD‐like receptor thermoprotein domain 3
SCI: spinal cord injury
TNF‐α: tumor necrosis factor‐α
UDCA: ursodeoxycholic acid
WB: Western blotting

## 1 Introduction

Spinal cord injury (SCI) represents a devastating traumatic disorder characterized by irreversible degeneration within the central nervous system (CNS). This pathological progression frequently culminates in profound and lasting functional deficits and, in its most severe forms, can be life‐threatening [1]. Cervical SCI is the predominant form of SCI, accounting for approximately 60% of all cases. The fifth cervical segment (C5) is most frequently affected [2], often leading to partial or complete loss of sensory and/or motor function below the site of injury. The pathological progression of SCI typically unfolds through two discrete yet interlinked phases: the immediate mechanical insult (primary injury) and the subsequent pathological cascade (secondary injury). The primary injury is characterized by irreversible pathological damage, whereas the secondary injury, developing on its basis, exacerbates tissue destruction and expands the affected area. This process involves multiple complex pathophysiological mechanisms, including immune activation and inflammation [3], which may serve as promising therapeutic targets for SCI. Given the grim prognosis of SCI and the absence of effective, practical interventions, clarifying the mechanisms driving secondary injury, such as inflammation is essential for advancing therapeutic strategies.

Inflammasomes are supramolecular organizing centers of the innate immune system [4]. These cytoplasmic multiprotein complexes assemble upon detection of pathogen‐associated (PAMPs) or damage‐associated molecular patterns (DAMPs), thereby playing a critical role in orchestrating the host's defensive responses [5, 6]. However, when pathological conditions such as tissue injury trigger abrupt and uncontrolled cell death, they can provoke abnormal immune reactions and excessive programmed cell demise. Pyroptosis, a distinct form of cell death initiated by Nod‐like receptor protein 3 (NLRP3) inflammasome activation, is intricately linked to the pathological progression of various neurological disorders, including SCI [7, 8]. The NLRP3 inflammasome, expressed predominantly in microglia—the resident macrophages of the CNS—serves a pivotal role in coordinating neuroimmune responses. Its core structure comprises the NLRP3 scaffold, the ASC adaptor, and pro‐Caspase‐1 (Caspase‐1 precursor) [9]. Under homeostatic conditions, intramolecular domain interactions maintain NLRP3 in a quiescent, auto‐repressed state. When exposed to stressors such as PAMPs or DAMPs, this equilibrium is disrupted [9, 10], triggering conformational changes and cleavage of NLRP3. These events promote the recruitment and activation of pro‑Caspase‑1. The activated Caspase‐1 then catalyzes the maturation of pro‐IL‐1β and pro‐IL‐18 into their bioactive forms, IL‐1β and IL‐18, thereby enabling their secretion and amplifying the inflammation. Meanwhile, this signaling cascade mediates the proteolytic processing of gasdermin D (GSDMD), a key downstream effector. The liberated N‐terminal domains form pores in the plasma membrane, ultimately precipitating the execution of pyroptosis [11, 12]. The process of pyroptosis consequently manifests as membrane disruption and the release of cytoplasmic contents, including proinflammatory cytokines [13]. The NLRP3/Caspase‐1/GSDMD axis has been identified as a crucial mediator of secondary injury progression post‐SCI [14, 15]. On the one hand, substantial amounts of PAMPs and DAMPs are released both locally and systemically after SCI, stimulating the NLRP3 inflammasome [16] and upregulating related genes such as NLRP3, pro‐IL‐18, and pro‐IL‐1β [17]. This activation induces pyroptosis, leading to excessive cell death and a pronounced inflammation [18]; On the other hand, pyroptosis represents the loss and dysfunction of neurons, thereby intensifying the secondary damage associated with SCI [19]. In summary, targeting the NLRP3/Caspase‐1/GSDMD‐mediated pyroptosis with safe and effective interventions could prevent and control secondary injuries, including inflammatory responses, after SCI, thereby offering significant therapeutic benefits.

Ursodeoxycholic acid (UDCA), an endogenous secondary bile acid, represents the core bioactive constituent of bear bile‐related medications, which have a history of use in traditional Chinese medicine spanning millennia [20]. Clinically, modern purified or synthetic UDCA formulations are approved by the US Food and Drug Administration (FDA) for the management of specific cholestatic disorders [21]. Beyond its canonical hepatoprotective effects, accumulating evidence has demonstrated that UDCA possesses a spectrum of beneficial pharmacological properties, including roles in antioxidant, anti‐inflammatory, cytoprotective, and immunomodulatory processes [22, 23, 24]. Significantly, investigations into UDCA have extended to neurodegenerative conditions [25, 26]. In experimental settings, it has been documented to improve hindlimb motor performance in thoracic SCI rats [27], indicating preliminary promise. However, research on its neuroprotective effects in cervical SCI models and its role in improving forelimb motor function remains limited. In addition, evidence from prior studies indicates that UDCA could effectively inhibit the NLRP3 inflammasome, as observed in models of atherosclerosis and middle cerebral ischemia/reperfusion occlusion (MCAO), thereby alleviating inflammation and slowing lesion progression [28, 29]. These findings suggest that UDCA has potential to modulate the NLRP3/Caspase‐1/GSDMD pyroptotic signaling pathway in SCI; however, current research in this area remains scarce, and additional studies are required to validate these effects.

This study initially employed in vitro experiments to assess the impact of UDCA on lipopolysaccharide (LPS)‐induced NLRP3 protein expression in BV2 microglia, and offered a mechanistic basis for UDCA's potential to attenuate pyroptosis pathway activation. Subsequently, by establishing a rat model of acute C5 cervical SCI, the motor dysfunction and pathological changes in spinal cord tissues were systematically evaluated to elucidate the regulatory effect of UDCA on functional recovery and tissue repair. In parallel, the alterations in the expression of key components within the NLRP3/Caspase‐1/GSDMD axis and downstream inflammatory mediators were analyzed, providing molecular‐level evidence for the inhibitory effects of UDCA on inflammasome activation and its anti‐pyroptosis activity in SCI treatment.

## 2 Materials and Methods

### 2.1 Animals

Healthy adult male SPF‐grade Sprague‐Dawley rats weighing 240–290 g were procured from SiPeiFu (Beijing) Biotechnology Co. Ltd. (Animal Certificate No.: SCXK (Beijing) 2019–0010). To compensate for inevitable attrition resulting from anesthesia risk, surgical trauma, and postoperative complications, extra rats were reserved in advance. The animal facility was maintained in a quiet, clean environment with adequate ventilation and controlled humidity. To ensure animal welfare and experimental consistency, housing conditions were strictly controlled, including a temperature of 22°C ± 2°C, a 12‐h light‐dark cycle, and standard cage housing (480 × 350 × 200 mm, four rats per cage). Food and water were provided ad libitum, and the bedding was replaced every 2 days. Before SCI modeling and intervention, all rats underwent a 7‑day acclimation period to minimize potential bias from environmental adaptation. The study was approved by the Ethics Committee for Animal Experiments of the General Hospital of Western Theater Command (Ethical approval number: 2024EC7‐ky033).

### 2.2 Materials

UDCA was acquired from Proteintech Group, Inc (Wuhan, China). LPS (derived from Escherichia coli 055:B5) was sourced from Wuhan Servicebio Technology Co., Ltd (Wuhan, China). Cell Counting Kit (CCK)‐8 for the detection of BV2 cell viability was procured from Beijing Labgic Technology Co., Ltd (Beijing, China). Primary anti‐NLRP3 antibody (bs‐10021R) was obtained from Bioss Biotechnology Co., Ltd (Beijing, China). Primary antibodies against Caspase‐1 (ab179515) and GSDMD (ab219800) were obtained from Abcam (Cambridge, UK). Anti‐Iba‐1 primary antibody (ET1705‐78) was obtained from HUABIO (Hangzhou, China). GAPDH (10494‐1‐AP) and Vinculin (26520‐1‐AP) Polyclonal antibody were purchased from Proteintech Group, Inc (Wuhan, China). The Horseradish Peroxidase (HRP)‐conjugated goat anti‐rabbit IgG (SA00001‐2) and 647‐goat anti‐rabbit recombinant secondary antibody (RGAR005) were obtained from Proteintech Group, Inc (Wuhan, China). The Fluorescein Isothiocyanate (FITC)‐conjugated goat anti‐rabbit IgG (GB22303) was obtained from Wuhan Servicebio Technology Co., Ltd (Wuhan, China). ELISA kits for IL‑18 and IL‑1β were supplied by Elabscience Biotechnology Co., Ltd (Wuhan, China), while the tumor necrosis factor‑α (TNF‑α) assay kit was purchased from NeoBioscience Technology Co., Ltd (Shenzhen, China).

### 2.3 Experimental Protocol

After a 7‐day period of feeding and adaptation, the rats were randomly assigned to one of three groups using a random number table before surgery: the Sham Surgery group (Sham), the SCI group, and the UDCA Intervention group (SCI + UDCA). The SCI and SCI + UDCA groups underwent cervical level 5 semi‐lateral clamp SCI induction using a medical aneurysm clip applying a clamping force of 70 g. Preoperative body weights were measured to determine the precise anesthetic dosage. Rats were anesthetized with an intraperitoneal injection of 1.5% pentobarbital sodium (0.33 mL/100 g). Thereafter, a midline dorsal incision approximately 5 cm in length was executed, extending cranially from the T2 spinous process as the anatomical landmark. The tissue was carefully dissected layer by layer and C5 laminectomy was performed (Figure 1A). After fully exposing the spinal cord (focusing on the right side), the aneurysm clip was spread open and slowly inserted into the spinal cord tissue at the C5 segment from the right. Once its tip reached half the transverse diameter of the spinal cord and made contact with the central vein, the aneurysm clip was released to apply compression for 30 s. The instrument was then removed, and the tissues were sutured layer by layer in accordance with anatomical structures. To counteract potential dehydration, all animals received a daily intraperitoneal injection of 5 mL of physiological saline for three consecutive days postoperatively. The model validation indicators included a characteristic flexion deformity in the affected forelimbs of rats. During spontaneous movement, the rats tended to tilt toward the affected side, with the dorsal surface of the affected forepaw abnormally contacting the ground; When the tail was lifted to suspend the rat, the affected limb showed impaired extension and was unable to perform normal forward and downward stretching movements (Figure 1B). In the Sham group, rats underwent only vertebral lamina removal without spinal cord clamping, preserving the integrity of tissues, while all other procedures were identical to those performed in the injury groups. Minor physiological attrition occurred throughout the experimental period due to SCI‐related surgical complications, which is typical for traumatic spinal cord lesion models. To ensure balanced sample size and consistent baseline conditions across three groups, 12 qualified surviving rats per group were finally confirmed and included for subsequent assessments. No animals were excluded selectively according to other reasons.

**Figure 1:**
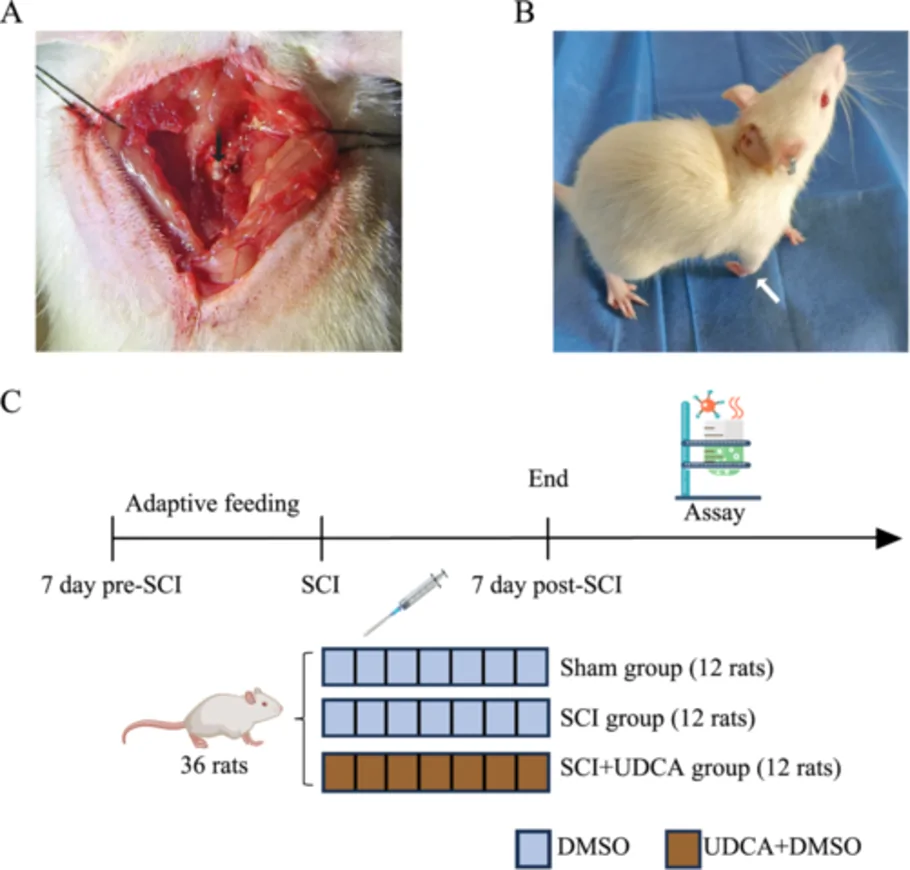
Spinal cord injury modeling and experimental design. (A) C5 laminectomy and spinal cord exposure. (B) The affected upper limb of the cervical SCI rat displays significant functional impairment after modeling. (C) Rats in the SCI + UDCA group received daily oral UDCA dissolved in 1% (v/v) DMSO postmodeling, while the SCI and Sham groups were administered 1% (v/v) DMSO alone. The intervention was carried out for 7 consecutive days.

Following the modeling procedure, UDCA was formulated for oral gavage according to previously reported protocols for in vivo studies. Briefly, UDCA was first dissolved in DMSO to prepare a 250 mg/mL stock solution, which was further diluted with sterile 0.9% saline to yield a working solution containing 1% (v/v) DMSO before administration. Rats in the SCI + UDCA group received UDCA at a daily dose of 25 mg/kg [27] via oral gavage starting immediately after surgery. Parallel administration of an equal volume of vehicle (1% DMSO in 0.9% saline) was performed in the Sham and SCI groups. All rats were given free access to food and water postsurgery, and the entire intervention period lasted for 7 days (Figure 1C). Upon completion of the 7‐day intervention, rats were deeply anesthetized and subsequently euthanized. Blood samples and spinal cord tissues were harvested for assessment of serum biomarkers and tissue protein expression levels.

Before tissue collection, animals within each group (*n* = 12) were randomly assigned to independent subsets for different analyses, as tissue obtained from one individual cannot be utilized for all endpoint analysis. Specifically, animals undergoing transcardial perfusion with paraformaldehyde yield fixed spinal tissues suitable for HE staining and immunofluorescence, yet these fixed specimens are incompatible with Western blotting. Accordingly, six rats from each group were randomly allocated for behavioral testing and Western blot detection, and the remaining animals in each group were subjected to histological staining, immunofluorescence assays, and ELISA measurements. This design also avoids potential bias arising from repeated measurement of indicators derived from the same animal.

### 2.4 Behavioral Evaluation and Motor Function Assessment

Behavioral assessment of forelimb motor function was conducted using the Cylinder Rearing test before modeling (baseline) and on postoperative Days 1, 3, 5, and 7. The Cylinder Rearing test is a well‐established method for evaluating forelimb motor dysfunction in rats [30]. In this test, rats are placed in a cylindrical chamber, and the frequency of forelimb contacts with the chamber wall is recorded to determine the usage rate of the injured forelimb. The calculation formula is as follows: total number of wall contacts by the affected forelimb/(number of independent wall contacts by the affected forelimb + number of independent wall contacts by the contralateral forelimb + number of joint wall contacts by both forelimbs). The total wall contacts by the affected forelimb were determined by adding the number of its independent contacts to half the number of joint contacts made by both forelimbs.

During the experiment, a transparent acrylic cylinder (20 cm diameter, 30 cm height) served as the testing arena. To ensure optimal viewing, specially angled mirrors were arranged bilaterally to permit comprehensive behavioral monitoring (Figure 3C). High‐definition video equipment was mounted on a stand, with the lens aligned to the cylinder's longitudinal axis, to capture the entire experiment and clearly record both the cylinder and the mirror reflections on either side. Each rat was recorded for approximately 15 min. Finally, the recorded sessions were scored by evaluators blinded to the group allocation using predefined criteria to quantify the forelimb usage rate.

### 2.5 Hematoxylin‐Eosin Staining

Spinal cord tissues harvested after perfusion underwent routine histological processing, including dehydration, paraffin embedding, and sectioning. To prepare for staining, tissue sections underwent deparaffinization through sequential 10‐min incubations in xylene I and II, followed by incubation in graded ethanol solutions (100%, 95%, 85%, 75%) for 5 min each. Sections were then stained according to the following protocol: (1) hematoxylin immersion for 5 min; (2) differentiate in differentiation solution for 30 s; (3) soak in bluing solution for approximately 1 min; (4) counterstain with eosin for 5 min. Slides were rinsed with running water after each step. After staining, the slides underwent reverse gradient ethanol dehydration (75%, 85%, 95%, and 100%) and were cleared sequentially in xylene I and II. Finally, they were mounted using neutral resin. Systematic examination of the stained slides was performed by unaffiliated researchers using a histopathology microscope integrated with a high‐resolution image capture system. Histopathological features were assessed at multiple magnifications, with representative fields of view carefully documented. The lesion cavity size was determined through morphometric analysis performed with ImageJ software.

### 2.6 Western Blot Analysis

Spinal cord tissue samples were homogenized and total protein was extracted in strict adherence to the guidelines provided with the protein extraction kit (Solarbio, Beijing, China). The resulting protein concentrations were then measured using the Bicinchoninic acid (BCA) method. Equal quantities of total protein were loaded onto precast gels, resolved by 10% sodium dodecyl sulfate ‐ polyacrylamide gel electrophoresis (SDS‐PAGE) (80 V for 40 min, followed by 160 V for 1 h), and subsequently transferred to PVDF membranes. Following transfer, the membranes underwent a blocking step with 5% nonfat milk for 1 h at room temperature. They were then incubated overnight at 4°C with primary antibodies targeting NLRP3 (1:1000, bs‐10021R, Bioss), Caspase‐1 (1:1000, ab179515, Abcam), GSDMD (1:1000, ab219800, Abcam), GAPDH (1:5000, 10494‐1‐AP, Proteintech), and Vinculin (1:5000, 26520‐1‐AP, Proteintech). Following primary antibody incubation, membranes were probed with HRP‐conjugated secondary antibodies (1:5000, SA00001‐2, Proteintech) for 1 h at ambient temperature. Protein bands were visualized by applying an ECL reagent and imaging with an Azure Biosystem instrument. The relative grayscale values of the bands were determined using ImageJ.

### 2.7 Enzyme‐Linked Immunosorbent Assay (ELISA)

Serum concentrations of IL‐18 (E‐EL‐R0567, Elabscience), IL‐1β (E‐EL‐R0012, Elabscience), and TNF‐α (ERC102a, NeoBioscience) were quantified using ELISA kits specific for rats, in strict accordance with the manufacturer's protocols. A typical procedure is as follows.

All samples, diluted standards, and blank controls were assayed in duplicate following a predefined plate layout. Specifically, 100 μL of each solution was pipetted into the appropriate wells. Following the initial incubation at 37°C for 90 min, each well was supplemented with 100 μL of the biotinylated detection antibody working solution. The plate was then incubated again at 37°C for 60 min. Following aspiration, each well was rinsed with 350 μL of wash buffer. Next, 100 μL of HRP conjugate working solution was added, and the plate was incubated at 37°C for 30 min before a final series of washes. Color development was initiated by adding 90 μL of Substrate Reagent to each well, followed by a 15‐min incubation at 37°C in the dark. The color reaction was subsequently quenched by the addition of 50 μL stop solution. The optical density (OD value) at 450 nm was determined for all wells using a microplate reader. Sample concentrations were calculated based on a standard curve derived from the reference standards.

### 2.8 Cell Culture and Treatment

The BV2 cells (official cell line name: BV2 microglia cell line, RRID: CVCL_0182, product number: CL‐0493) were purchased from Procell Life Science & Technology Co., Ltd (Wuhan, China) in October 2024. This cell line was immortalized from the cerebral cortex tissue of female C57BL/6 mice with a definite species and tissue origin. Before delivery, the cell line was verified and confirmed to be free of contamination by the supplier. This cell line has been extensively authenticated in previous in vitro studies investigating neural mechanisms of SCI recovery [31, 32] and NLRP3 inflammasome activation [33, 34], with no prior records of misidentification or contamination. Furthermore, all cell culture and experimental operations in the present study were performed in strict accordance with standard aseptic protocols. The culture environment, consumables, and medium were strictly sterilized and monitored throughout the experiments to prevent exogenous contamination, ensuring the mycoplasma‐free status of the cells used in this study. Standard culture conditions consisted of Dulbecco's Modified Eagle Medium (DMEM medium) enriched with 10% fetal bovine serum and 1% penicillin/streptomycin, incubated at 37°C in a 5% CO_2_. Cells were harvested during the logarithmic growth phase and either subcultured at a 1:3 ratio or seeded for subsequent experiments [35]. In this experiment, mouse BV2 microglia were allocated into three groups under distinct culture conditions: (1) Blank control group (Control): Cells were routinely cultured in standard complete medium; (2) LPS stimulation group (LPS): Log‐phase BV2 cells were treated with medium containing 100 ng/mL LPS for 24 h; (3) UDCA intervention group (LPS + UDCA): Based on CCK‐8 assay results and previous literature, cells were preincubated with 50 μmol/L UDCA for 12 h and subsequently stimulated with 100 ng/mL LPS for an additional 24 h [36, 37].

### 2.9 Cell Counting Kit‐8 Assay

BV2 cells in the logarithmic growth phase were suspended, and 100 μL of the cell suspension (5 × 10^4^ cells/mL) was distributed into individual wells of a 96‐well plate to achieve uniform plating. The experiment included a control group (medium only, UDCA concentration 0 μmol/L) and treatment groups exposed to varying UDCA concentrations (12.5, 25, 50, 100 μmol/L) to identify the optimal drug concentration range. Additionally, combined treatment groups were established by co‐administering different concentrations of UDCA with LPS. To be specific, cells were preincubated for 12 h in medium containing varying UDCA concentrations, followed by a 24‐h culture in medium supplemented with 100 ng/mL LPS, to observe changes in cellular activity induced by different drug concentrations and LPS co‐exposure. All experiments were performed in duplicate wells. Cell viability was assessed using a Cell Counting Kit‐8 (Biosharp) according to the manufacturer's instructions. The OD value at 450 nm was quantified with a microplate reader (ThermoFisher Scientific, USA). The calculation formula is as follows: Viability (%) = [(OD_treat_ − OD_Blank_)/(OD_Control_ − OD_Blank_)] × 100%.

### 2.10 Immunofluorescence

Immunofluorescence staining was performed on both cell coverslips and paraffin‐embedded rat spinal cord sections.

A 2 mL aliquot of cell suspension was seeded onto coverslips in 6‐well plates, and the culture conditions for each cell group were strictly maintained according to the predefined experimental protocol. Upon completion of the treatment, the culture supernatant was aspirated from each well, and the cells were exposed to a membrane‐permeabilizing solution for 20 min at room temperature. Subsequently, a blocking step was performed using 3% BSA solution for 30 min. Specific fluorescence labeling was achieved through sequential incubations: first with an anti‐NLRP3 primary antibody (1:200, bs‐10021R, Bioss) at 4°C overnight, followed by a FITC‐conjugated goat anti‐rabbit IgG secondary antibody (1:200, GB22303, Servicebio) for 50 min at room temperature. For nuclear visualization, DAPI staining was performed. After mounting with an anti‐fluorescence quenching medium, images were captured using a Nikon ECLIPSE CI‐L microscope.

For paraffin‐embedded spinal cord tissue: tissues were cut into 3 μm sections. Sections were deparaffinized in xylene, rehydrated through a graded ethanol series, and rinsed in phosphate buffer saline (PBS). Antigen retrieval was performed before staining. Sections were incubated overnight at 4°C in a humidified chamber with anti‐Iba‐1 primary antibody (1:500, ET1705‐78, HUABIO). On the following day, after PBS washing, sections were incubated with a 647‐goat anti‐rabbit recombinant secondary antibody (1:500, RGAR005, Proteintech) for 60 min at room temperature. After further PBS rinsing, nuclei were stained with DAPI solution in a humidified chamber for 10 min. Sections were incubated with an autofluorescence quencher for 5 min to reduce tissue endogenous autofluorescence, followed by mounting with anti‐fluorescence quenching medium. All images were acquired with the fluorescence microscope, and fluorescence intensity was quantified using ImageJ software.

### 2.11 Statistical Analysis

Statistical analyses were carried out using SPSS version 25.0 (SPSS Inc., Chicago, IL, USA). Continuous variables are reported as mean ± SD. The normality of distribution and homogeneity of variances were verified before analysis. Depending on these results, either one‐way ANOVA with LSD post hoc test or the nonparametric Kruskal–Wallis test followed by Mann–Whitney *U*‐tests were appropriately applied. The threshold for statistical significance was defined as *p* < 0.05.

## 3 Results

### 3.1 Appropriate Concentration of UDCA Could Enhance BV2 Cell Viability

CCK‐8 assay results indicated that BV2 cell viability exhibited a general pattern of initial increase followed by decline. This trend was consistent under both UDCA monotherapy and UDCA‐LPS co‐treatment conditions (Figure 2A) (Supporting Information: Tables 1 and 2). Notably, in the UDCA monotherapy experiments, cell viability was significantly suppressed (*p* < 0.01) when the UDCA concentration reached 100 μmol/L, demonstrating a statistically marked decline relative to the control group (0 μmol/L, *p* < 0.05). In the combined UDCA and LPS treatment experiments, BV2 cell viability showed no statistically significant differences across varying UDCA concentrations. Nonetheless, it exhibited a trend of initial increase followed by a decline, reaching its lowest level at 100 μmol/L. Consequently, 50 μmol/L UDCA was identified as the working concentration for this study to sustain the growth and vitality of BV2 microglia.

**Figure 2:**
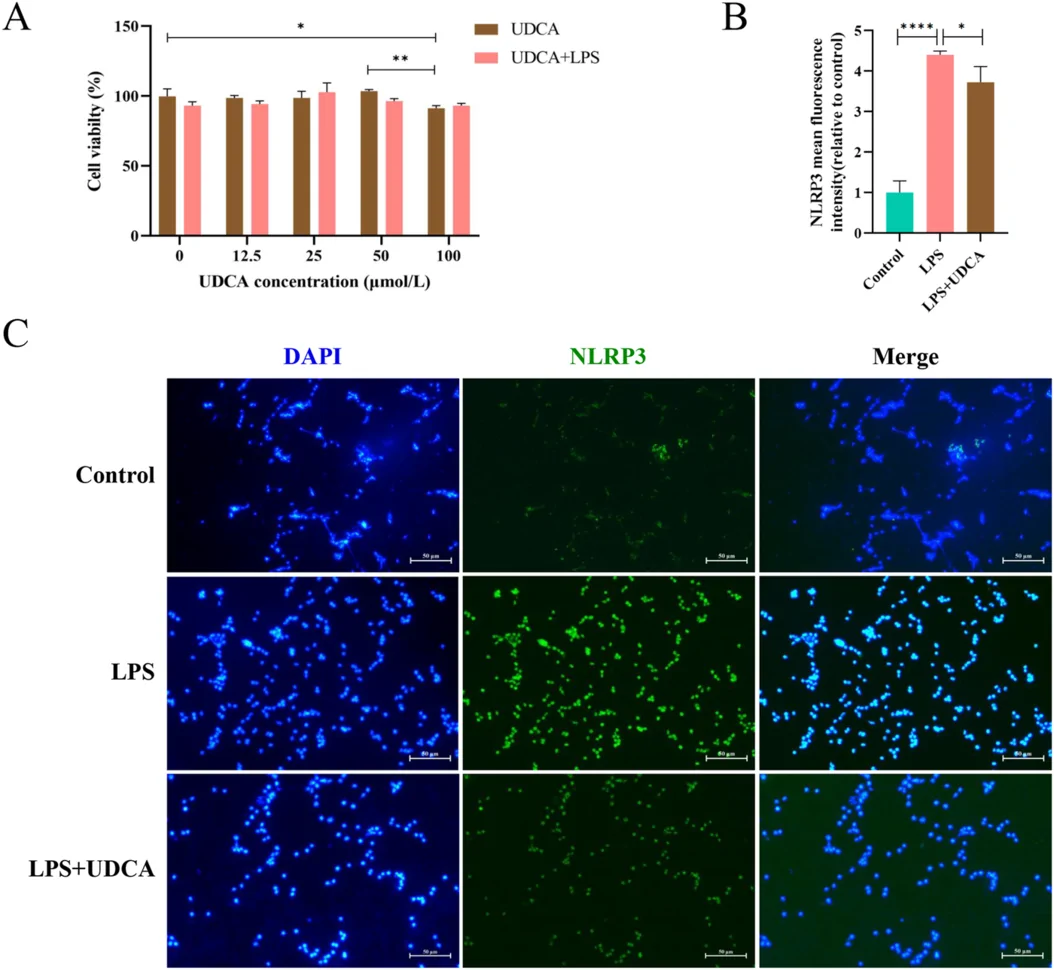
Appropriate concentration of UDCA enhances BV2 cell viability and suppress NLRP3 expression. (A) Effects of varying UDCA concentrations, alone or combined with LPS treatment, on BV2 cell viability. (B, C) The expression of NLRP3 (green) was analyzed by immunofluorescence. Nuclear chromatin was stained with DAPI (blue). Scale bar = 50 μm. Data are presented as mean ± SD. *p < 0.05, **p < 0.01, ****p < 0.0001.

### 3.2 UDCA Inhibits LPS‐Induced NLRP3 Expression in BV2 Cells

Adequate NLRP3 inflammasome activation is a crucial prerequisite for triggering the pyroptosis pathway and downstream inflammation. Therefore, to evaluate the inhibitory capacity of UDCA on NLRP3 expression in BV2 cells, this study employed immunofluorescence analysis to quantitatively assess changes in NLRP3 inflammasome expression (Figure 2B,C). Quantitative analysis demonstrated a robust upregulation of the fluorescence signal intensity of NLRP3 in BV2 cells following LPS challenge relative to the control group (*p* < 0.0001). Conversely, co‐treatment with UDCA markedly reduced NLRP3 fluorescence intensity relative to the LPS‐only group (*p* < 0.05) (Supporting Information: Table S3). The results suggest that UDCA effectively inhibits the LPS‐induced upregulation of NLRP3 expression in BV2 cells.

### 3.3 UDCA Promotes Recovery From Pathological Damage and Improves Fore Limb Motor Function in Acute Cervical SCI Rats

HE staining was conducted to systematically evaluate the histopathological characteristics of spinal cord tissue damage in each rat group (Figure 3A). Observations in the sham group revealed well‐preserved spinal cord integrity, with a distinct demarcation between gray and white matter and no evident pathological alterations. The SCI group showed pronounced disruption of spinal cord architecture, characterized by extensive cystic cavity formation and significantly reduced neuronal density. In contrast, the UDCA‐treated group exhibited attenuated tissue damage, smaller cavity areas, and partial restoration of structural integrity. Additionally, ImageJ software was employed to analyze HE‐stained sections and quantify the proportion of cavity areas (Figure 3B) (Supporting Information: Table S4). Comparative analysis among groups revealed that the sham surgery group maintained normal cavitation levels, whereas the SCI group exhibited a significant increase in cavitation. In contrast, UDCA treatment significantly reduced the percentage of cavitation areas (*p* < 0.01). These quantitative findings indicate that UDCA treatment effectively mitigates pathological progression in spinal cord tissue following SCI.

**Figure 3:**
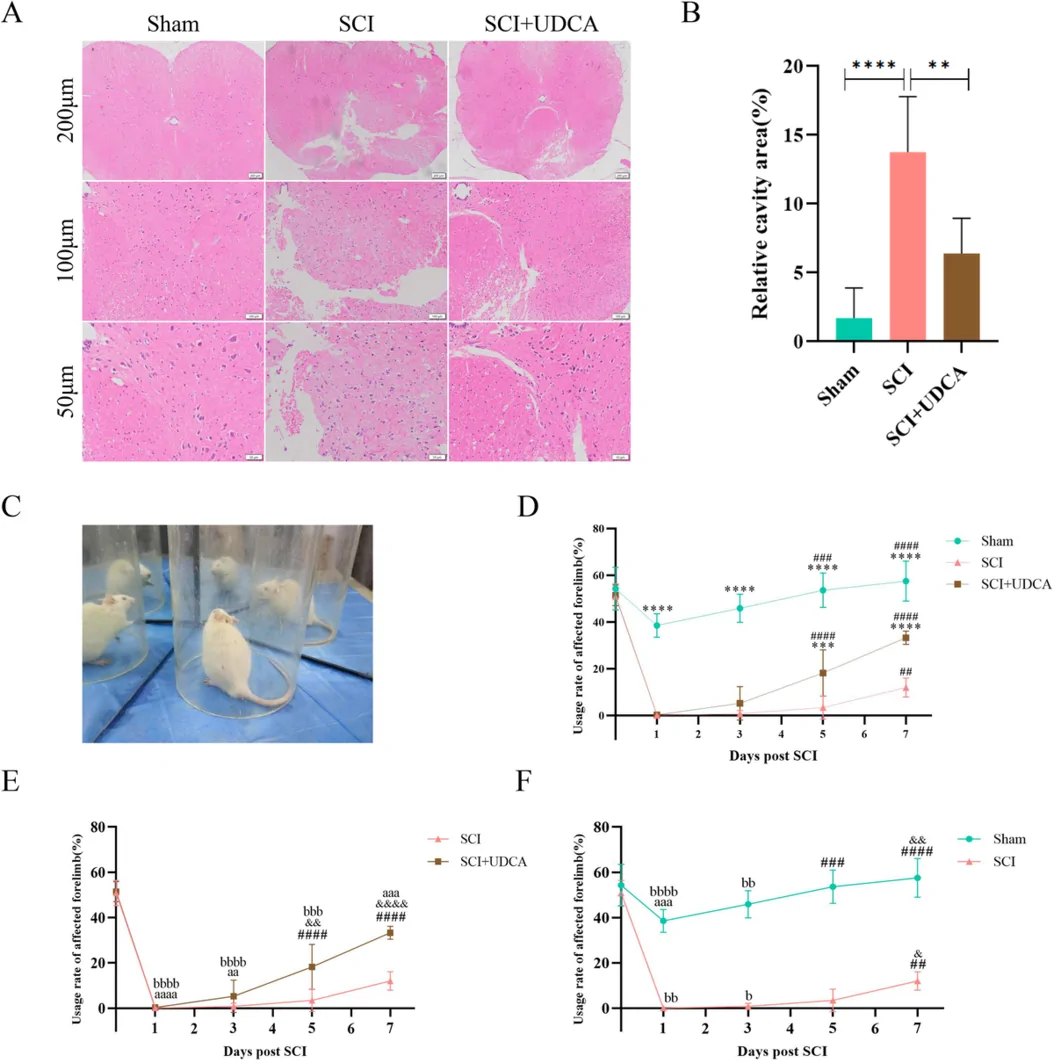
UDCA alleviates pathological damage in rats with acute C5 cervical SCI and promotes recovery of forelimb motor function. (A) Representative HE‐stained histopathological images of the spinal cord from each rat group. (B) Quantitative assessment of the relative cavity area in spinal cord tissue across groups, *n* = 6 for each group. (C) Schematic illustration of the Cylinder Rearing Test. (D–F) Quantitative evaluation of the functional status of the affected forelimb in each group at various observation time points. The usage rate of the affected forelimb was calculated as: total number of wall contacts by the affected forelimb/(number of independent wall contacts by the affected forelimb + number of independent wall contacts by the contralateral forelimb + number of joint wall contacts by both forelimbs). *n* = 6 for each group. Data are presented as mean ± SD. Compared with the SCI group at the same postoperative time point, **p < 0.01, ***p < 0.001, ****p < 0.0001. Compared with the results obtained 1 day postoperatively within the same group, ^##^ *p* < 0.01, ^###^ *p* < 0.001, ^####^ *p* < 0.0001. Compared with the results obtained 3 days postoperatively within the same group, ^&^ *p* < 0.05, ^& &^ *p* < 0.01, ^& & & &^ *p* < 0.0001. Compared with the results obtained 5 day postoperatively within the same group, ^aa^ *p* < 0.01, ^aaa^ *p* < 0.001, ^aaaa^ *p* < 0.0001. Compared with the results obtained 7 day postoperatively within the same group, ^b^ *p* < 0.05, ^bb^ *p* < 0.01, ^bbb^ *p* < 0.001, ^bbbb^ *p* < 0.0001.

The Cylinder Rearing test further validated the therapeutic efficacy of UDCA treatment in improving forelimb motor dysfunction in rats with acute C5 cervical SCI (Figure 3C–F) (Table S5). The results indicated that, before surgery, all three groups exhibited similarly high wall‐touching frequencies of the affected limb, as evidenced by statistically indistinguishable affected forelimb usage rates (*p* > 0.05). On Day 1 following the surgical procedure, during the acute postoperative phase, both the SCI model group and the UDCA treatment group displayed marked motor compensatory behavior, characterized by predominant reliance on the contralateral forelimbs and a sharp decline in use of the affected limbs (*p* < 0.0001). In the Sham group, rats also exhibited a significant drop in scores on the first postprocedural day (*p* < 0.001); however, by Day 3 postmodeling, their scores had largely recovered to preoperative levels. On Days 3, 5, and 7 after modeling, the usage rate of injured forelimbs increased across all groups, accompanied by progressive improvement in Cylinder Rearing performance. Throughout the observation period, rats in both the SCI and SCI + UDCA groups demonstrated sustained impairment in motor function, with behavioral scores remaining significantly lower than those recorded in the Sham group (Figure 3D). The temporal pattern of functional recovery differed notably between groups. While a significant improvement in the SCI group was not observed until Day 7 (*p* < 0.01) (Figure 3F), the SCI + UDCA group displayed a pronounced and early improvement that reached statistical significance by Day 5 (*p* < 0.0001) (Figure 3E), suggesting that UDCA treatment markedly accelerated motor recovery in acute cervical SCI rats. Intergroup comparisons revealed that rats in the SCI + UDCA group demonstrated superior recovery of forelimb motor function at all postoperative time points relative to the SCI group. Their Cylinder Rearing test scores were consistently higher, with statistical significance emerging by Day 5 postmodeling (*p* < 0.001) (Figure 3D). These findings indicate that UDCA treatment may improve ipsilateral forelimb utilization in acute cervical SCI rats and facilitate the restoration of forelimb motor function.

### 3.4 UDCA Suppresses the Expression of Essential Components of the NLRP3/Caspase‐1/GSDMD Pyroptosis Signaling Cascade in Rats With Acute Cervical SCI

Western blotting confirmed the activation of the pyroptosis pathway in spinal cord tissue after injury, as evidenced by the elevated protein levels of pyroptosis‐related key components, NLRP3, GSDMD, and N‐GSDMD, in the SCI group versus the Sham group (*p* < 0.05), accompanied by an upward trend in Caspase‐1 expression. UDCA intervention significantly suppressed NLRP3 inflammasome activation (*p* < 0.05), markedly downregulated GSDMD expression (*p* < 0.001), and controlled Caspase‐1 protein activation as well as N‐GSDMD formation (Figure 4A–F) (Supporting Information: Table S6). These collective findings suggest that UDCA treatment could partially counteract SCI‐induced pyroptosis pathway activation and suppress the overexpression of its key proteins.

**Figure 4:**
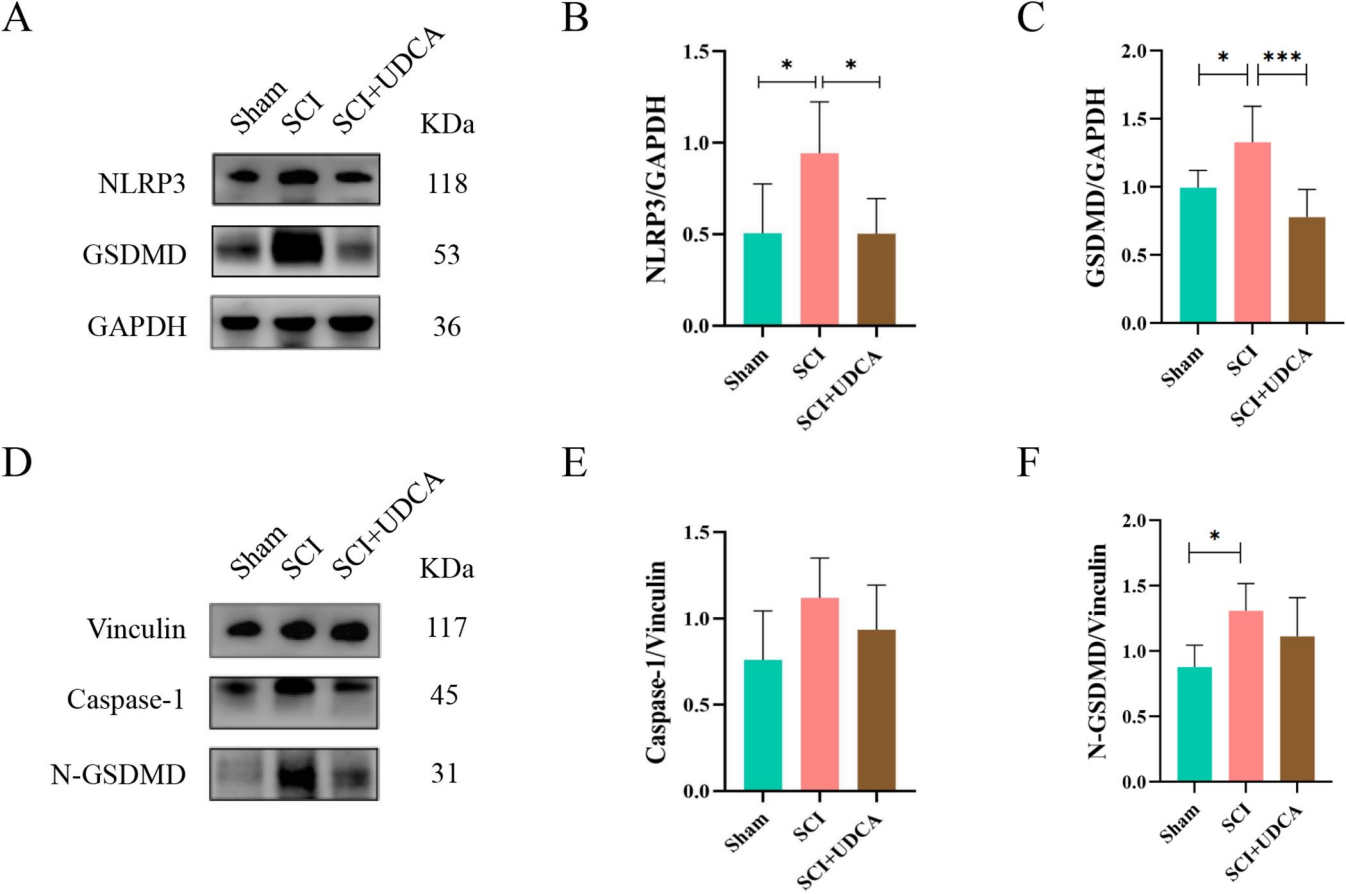
UDCA suppresses the NLRP3/Caspase‐1/GSDMD pyroptosis signaling cascade in rats with acute C5 cervical SCI. (A) Representative Western blot images showing the expression of NLRP3 and GSDMD in spinal cord tissues. (B, C) Quantitative analysis of NLRP3 and GSDMD protein expression in spinal cord tissues, with GAPDH serving as internal controls. *n* = 6 for each group. (D) Representative Western blot images showing the expression of Caspase‐1 and N‐GSDMD in spinal cord tissues. (E, F) Quantitative analysis of Caspase‐1 and N‐GSDMD protein expression in spinal cord tissues, with Vinculin serving as internal controls. *n* = 6 for each group. Data are presented as mean ± SD. *p < 0.05, ***p < 0.001.

### 3.5 UDCA Attenuates SCI‐Induced Local Tissue and Systemic Inflammation

To further characterize UDCA's anti‐pyroptotic and anti‐inflammatory effects after SCI, immunofluorescence staining and ELISA assays were carried out. Immunofluorescence was applied to assess Iba‑1 (microglial marker) expression adjacent to the SCI lesion (Figure 5A). Iba‑1 immunoreactivity was significantly upregulated in the SCI group versus the Sham group (*p* < 0.001), and UDCA treatment lowered Iba‑1 signals (*p* < 0.05) (Figure 5B) (Table S7).

**Figure 5:**
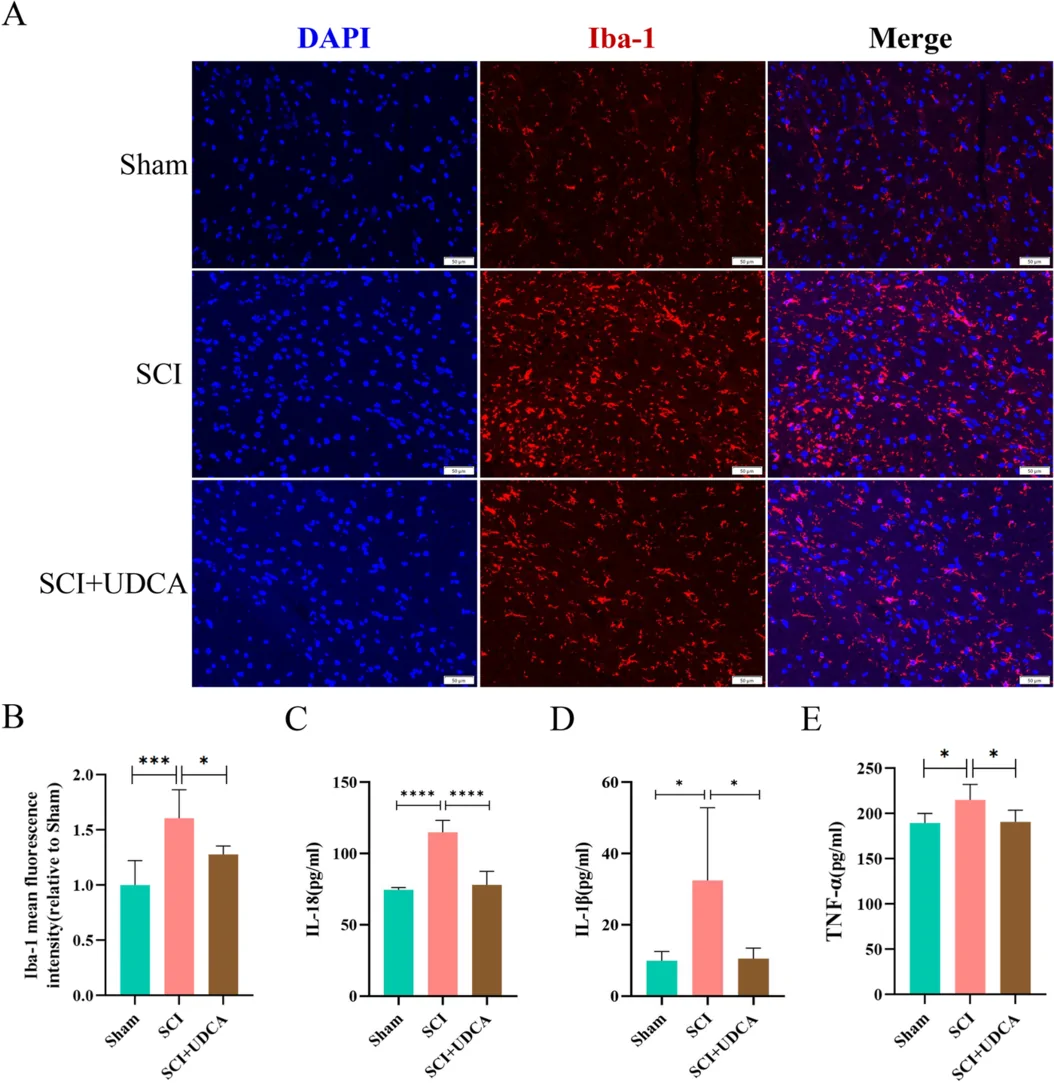
UDCA attenuates SCI‐induced local tissue and systemic inflammation. (A) Immunofluorescence staining of Iba‐1 (red) in region C5 of the spinal cord. Scale bar = 50 μm. (B) Comparison of relative fluorescence intensity of spinal cord Iba‐1. *n* = 6 for each group. (C–E) Serum levels of IL‐18, IL‐1β, and TNF‑α evaluated by ELISA in three groups of rats, *n* = 5 for each group. Data are presented as mean ± SD. *p < 0.05, ***p < 0.001, ****p < 0.0001.

Serum levels of pyroptosis‐associated IL‑18 and IL‑1β, as well as inflammatory TNF‑α, were quantified in all groups (Figure 5C–E) (Table S8). Compared with the Sham group, the SCI group showed markedly higher serum IL‑18 (*p* < 0.0001), IL‑1β, and TNF‑α (*p* < 0.05). UDCA intervention significantly reduced serum IL‑18 (*p* < 0.0001), as well as IL‑1β and TNF‑α levels relative to the SCI group (*p* < 0.05). These observations indicate that UDCA mitigates SCI‐induced tissue and systemic inflammation and reshapes the inflammatory profile.

## 4 Discussion

This study investigated the effects of the endogenous bile acid UDCA on neural pyroptosis and inflammation from the perspective of endogenous metabolites, exploring its regulatory mechanisms at cellular and animal levels. We preliminarily observed that UDCA modulates NLRP3 inflammasome activation in LPS‐stimulated BV2 cells and benefits functional recovery and tissue repair in a rat model of acute C5 cervical SCI. Furthermore, UDCA intervention correlated with reduced activation of the NLRP3/Caspase‐1/GSDMD pathway, alongside decreased secretion of IL‐18, IL‐1β, and TNF‑α postinjury, which may help attenuate secondary injury in this acute cervical SCI model (Figure 6).

**Figure 6:**
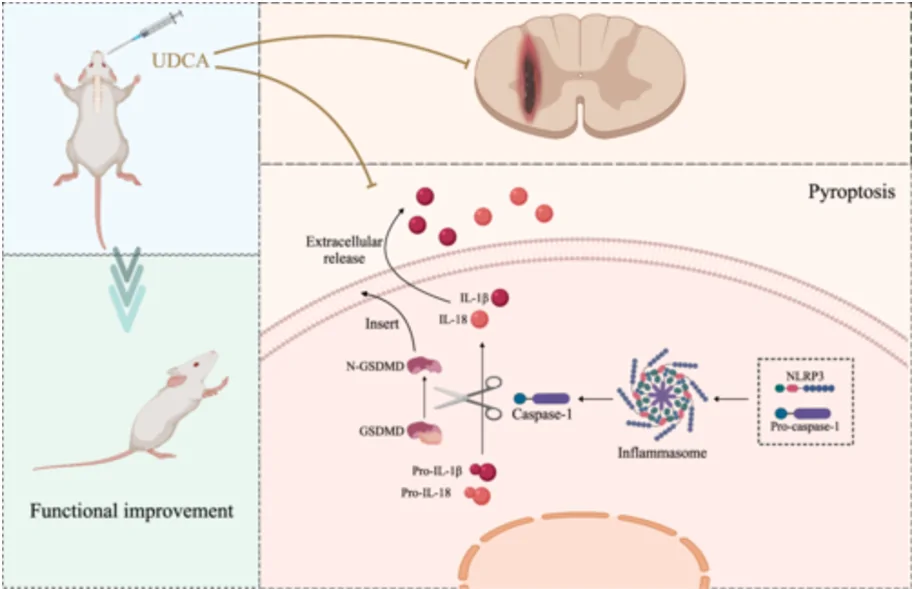
Potential mechanistic correlates of UDCA treatment. UDCA treatment is associated with altered activity of the NLRP3/Caspase‑1/GSDMD pyroptotic pathway and changes in the release of downstream inflammatory cytokines (IL‑18, IL‑1β) in rats with acute C5 cervical SCI.

Despite significant advances in medical interventions that have steadily improved the survival rates of patients with SCI [38], secondary pathological processes and resulting functional impairments continue to place a substantial burden on both patients and their families. Secondary injury refers to the cellular and biochemical consequences following primary damage, encompassing vascular rupture, hemorrhage, neuronal death, and inflammation [39, 40, 41]. In the acute phase, it is characterized by extensive infiltration of inflammatory cells—particularly microglia—around the lesion site [42]. As a key determinant of subsequent functional recovery after acute SCI, secondary injury mechanisms—such as inflammation—are complex yet, to some extent, reversible. Therefore, identifying safe and promising pharmacological candidates to enhance motor recovery, mitigate secondary injury, and clarify the underlying therapeutic mechanisms is of critical importance.

UDCA, an endogenous bile acid, represents a class of metabolites involved in gut‐brain axis interactions [43]. Notably, bear bile, a traditional Chinese medicine with UDCA as its major bioactive constituent, has been used for centuries [44], whereas modern purified or synthetic UDCA displays broad pharmacological activities and a favorable safety profile. Previous in vitro studies have confirmed that UDCA does not significantly affect cell viability. Even when macrophages were cultured with higher concentrations of UDCA, their viability remained at approximately 70%, suggesting a relatively mild effect of UDCA [45]. The demonstrated favorable safety and tolerability of UDCA in vivo further reinforce these findings [21]. Consistent with prior observations, this study confirms that UDCA, at appropriate concentrations, maintains and even enhances BV2 cell viability.

The neuroprotective potential of UDCA has attracted growing attention in recent years [46], with its therapeutic effects on thoracic SCI previously investigated. In rat models of T9 SCI, Ko et al. [27] and Kim et al. [47] demonstrated that UDCA treatment significantly improved hindlimb motor function recovery. This study further investigated the therapeutic potential of UDCA in alleviating forelimb motor dysfunction in a rat model of acute C5 cervical SCI, producing consistent outcomes. In C5 SCI rats treated with UDCA, performance in the Cylinder Rearing test was significantly improved, accompanied by greater utilization of the affected forelimbs and partial recovery of motor function. These findings suggest that UDCA exerts neuroprotective effects across various SCI segments and facilitates motor recovery. Additionally, HE staining revealed that UDCA treatment slowed pathological progression within the injured spinal cord, accompanied by partial restoration of compromised tissue integrity and reduced cavitation. Quantitative analysis further demonstrated that UDCA intervention preserved tissue architecture and restrained lesion expansion. In summary, these results indicate that UDCA may promote forelimb functional recovery in cervical SCI rats, attenuate pathological damage and disease progression, and confer potential therapeutic benefit.

Inflammasomes, particularly the NLRP3, serve as critical initiators of the pyroptosis pathway and inflammatory cascade [48], contributing to the pathological progression of various CNS disorders, including SCI [49, 50]. Microglial NLRP3 inflammasomes play a paradoxical dual role: they are essential for CNS immune surveillance and homeostasis, yet their maladaptive activation during acute injuries such as SCI constitutes a key pathological driver that exacerbates lesion progression. Such activation triggers pyroptosis, a distinct form of cell death that promotes excessive inflammation and secondary damage. Given the essential role of neuroinflammation control for recovery after degenerative and traumatic CNS disorders [11], modulating microglial inflammasome activity represents a promising therapeutic strategy. LPS derived from Gram‐negative bacteria is a potent immunostimulant, and its induction of BV2 microglia has become a classic model for simulating NLRP3 inflammasome activation under neuroinflammatory conditions [51]. Based on our in vitro findings, UDCA appears to attenuate NLRP3 inflammasome activation in LPS‐stimulated BV2 cells, indicating its potential to modulate pyroptosis‐associated signaling.

The NLRP3/Caspase‐1/GSDMD signaling axis, a well‐characterized pathway for pyroptosis execution, is tightly linked to secondary injury post‐SCI. Initially, pyroptosis was thought to be controlled by Caspase‐1 [52]; however, subsequent studies gradually revealed that GSDMD, activated through cleavage by Caspase‐1, serves as the primary executor of this process [53]. As NLRP3 inflammasome activation constitutes a core prerequisite for this canonical pyroptotic cascade [54], targeting NLRP3 inflammasome and the ensuing pyroptotic response may hold therapeutic potential against SCI. Notably, UDCA has been shown to bind and activate the G‑protein‑coupled bile acid receptor TGR5 (GPBAR1), which is reported to exert neuroprotective effects in various neurological disease models [55, 56]. Since accumulating preclinical evidence indicates that TGR5 signaling suppresses NLRP3 inflammasome assembly and activation [28], it is reasonable to hypothesize that UDCA may dampen NLRP3‐GSDMD‐mediated pyroptosis in vivo following SCI.

To extend our in vitro observations and further dissect UDCA's mechanisms in SCI, we examined the expression of core pyroptosis effectors in rat spinal cord tissue. In vivo data revealed that oral UDCA intervention reduced NLRP3 and GSDMD protein levels in the injured spinal cord, alongside a downward trend in Caspase‐1 activation and N‐GSDMD formation. Total GSDMD abundance reflects the cellular reservoir of this pyroptosis effector, while N‐GSDMD represents the proteolytically cleaved functionally active form driven by upstream caspase‑1 activation [57]. One plausible explanation for the discrepancy in our protein expression data is that UDCA predominantly suppresses the expression of full‑length GSDMD, thereby limiting the substrate pool available for subsequent cleavage into N‐GSDMD, rather than directly inhibiting caspase‑1‑dependent proteolytic cleavage of already‑existing GSDMD protein. At the 7‐day postinjury time point chosen for this acute C5 cervical SCI model, robust inflammasome activation and GSDMD cleavage had already been initiated by acute spinal cord trauma. A preclinical study reported that N‐GSDMD levels may peak within 3 days following SCI [58], such that preformed fragments could persist within the spinal cord parenchyma at Day 7 postinjury. Even though UDCA reduces de novo GSDMD synthesis within the observation window, preformed N‐GSDMD generated during the early acute phase persists within the tissue, which may account for the absence of a statistically significant difference in N‐GSDMD abundance between the SCI and SCI + UDCA groups. Such dissociation between total full‑length GSDMD and cleaved N‐GSDMD has been documented in previous in vivo research, indicating that GSDMD expression and its caspase‑1‑mediated cleavage can be regulated independently [59]. It should be noted that our findings cannot fully exclude time‑dependent dynamic changes of N‐GSDMD; earlier or later sampling time points may yield different responses to UDCA intervention.

Furthermore, immunofluorescence staining demonstrated that UDCA treatment reduced Iba‐1 immunoreactivity in the injured spinal cord, indicative of diminished microglial activation. Consistently, ELISA results revealed lower serum levels of IL‑18, IL‑1β, and TNF‑α in UDCA‐treated SCI rats. Microglia, the major resident immune cells in the spinal cord, act as key mediators of neuroinflammation after SCI [60]. Following injury, activated microglia secrete multiple pro‐inflammatory cytokines that amplify secondary spinal cord damage. These observations suggest that UDCA may alleviate both local spinal inflammation and systemic inflammatory responses in this acute C5 cervical SCI model. Considering the close association between microglial NLRP3 inflammasome activation, pyroptosis, and cytokine maturation, our findings imply a partial link between the anti‐inflammatory effects induced by UDCA and modulation of the NLRP3/Caspase‑1/GSDMD pyroptotic cascade after SCI.

The strengths of this study are summarized as follows. First, this work focuses on forelimb motor function, neural pyroptosis, and neuroinflammation after cervical SCI, and reveals an association between the NLRP3 inflammasome and pathological progression in an acute C5 cervical SCI model, supporting the potential of the NLRP3/Caspase‑1/GSDMD axis as a therapeutic target. Second, we combined in vitro BV2 microglial experiments and in vivo animal assays to characterize the correlation between UDCA intervention and changes in pyroptotic signaling and inflammatory responses. Third, this study adopted UDCA, an endogenous bile acid metabolite, as the intervention agent. Building on previous preclinical evidence regarding the safety profile of UDCA, our work provides insights into its biological effects in acute cervical SCI and helps unpack the potential mechanisms linked to its action. Collectively, these observations deepen our understanding of UDCA's preclinical potential and provide useful references for subsequent mechanistic investigations.

Although this study contributes meaningfully to the understanding of SCI treatment, several limitations should be acknowledged. First, our observations offer only preliminary insights into the association between UDCA and modulation of the NLRP3/Caspase‑1/GSDMD axis, as well as its neuroprotective effects in this acute C5 cervical SCI model. While these findings complement existing literature, the full mechanisms underlying UDCA's actions remain incompletely elucidated. Second, we only assessed pathway‐related protein changes at a single 7‐day postinjury time point; dynamic alterations of pyroptosis and neuroinflammation across earlier or later time windows were not characterized. Third, genetic or pharmacological inhibition assays were not performed to further dissect the causal linkage between the NLRP3/Caspase‑1/GSDMD cascade and the observed effects of UDCA. Furthermore, a potential challenge is that UDCA has low water solubility; therefore, DMSO was used as a cosolvent to dissolve UDCA for oral gavage in this study. Of note, DMSO itself may exhibit antioxidant and potential neuroprotective properties under certain experimental conditions [61, 62], as reported previously. In the present study, we adopted a low necessary DMSO concentration (1%, v/v) for solubilization to minimize such risks; such a dose of DMSO has been reported to be well‐tolerated in repeated administration without overt systemic toxicity or detectable confounding biological interference [63, 64, 65]. Solvent effects constitute a critical variable for in vivo pharmacological studies. While our current control design helps isolate the biological effects of UDCA, future investigations will evaluate alternative solvent formulations to further characterize potential solvent‐associated biological responses.

To further extend the findings of this experiment, we will continue to explore the potential of UDCA in acute C5 cervical SCI models. Planned investigations include collecting samples at multiple postinjury time points to characterize the dynamic changes in pyroptosis and neuroinflammation, varying the duration of UDCA administration, characterizing the drug's in vivo metabolic profile, and increasing sample size to identify additional molecular targets. These approaches will enable more comprehensive and precise characterization of UDCA‐associated biological effects. In addition, a fuller understanding of the onset and regulatory networks of secondary SCI injury remains essential. Although the present work focuses on the NLRP3 inflammasome and pyroptotic cascade, incorporation of additional biomarkers and genetic manipulation approaches, such as gene knockout may further clarify core mediators of secondary injury and support the development of refined intervention strategies. Lastly, the associations observed between UDCA treatment and changes in pyroptosis and inflammation within this preclinical model warrant further validation in follow‐up studies before any translational exploration.

## 5 Conclusions

In conclusion, combining in vitro BV2 microglial experiments and in vivo animal assessments, this study characterizes associations between UDCA intervention and attenuated secondary injury as well as improved functional outcomes in an acute C5 cervical SCI model. Our observations reveal that UDCA treatment is associated with lowered LPS‐induced NLRP3 expression in BV2 microglia. In rats with acute C5 cervical SCI, UDCA administration correlated with preserved forelimb motor function and alleviated spinal histopathological damage. Furthermore, our data point to a link between UDCA treatment and altered activity of the NLRP3/Caspase‑1/GSDMD cascade, accompanied by attenuated neural pyroptosis and neuroinflammation. Collectively, these findings provide experimental insights into UDCA's preclinical potential and may support further mechanistic research targeting acute cervical SCI, with implications for the development of novel preclinical intervention strategies.

## Author Contributions

**Honghu Song:** conceptualization, writing – original draft, formal analysis, methodology. **Xiaomin Hu:** conceptualization, writing – review and editing, funding acquisition. **Dan Liu:** methodology, data curation. **Linjie Wang:** visualization, software. **Li Zhu:** methodology, data curation. **Wenchun Wang:** validation, supervision. **Rongnan Hu:** data curation, investigation. **Jiayan Zhong:** investigation, software. **Jiancheng Liu:** writing – review and editing, formal analysis, funding acquisition. **Rizhao Pang:** project administration, pesources, funding acquisition. **Anren Zhang:** project administration, conceptualization, writing – review and editing, supervision.

## Ethics Statement

All the animal experiments procedures were performed with the approval of the Ethics Committee of the General Hospital of the Western Theater Command of the Chinese People's Liberation Army (No.2024EC7‐ky033). The study was conducted in accordance with the local legislation and institutional requirements.

## Conflicts of Interest

The authors declare no conflicts of interest.

## Supporting information


Supporting File

## Data Availability

The data that support the findings of this study are available in the supporting information of this article. The datasets used and/or analyzed during the current study are available from the corresponding author on reasonable request.
