# Supplementary material for: Ursodeoxycholic Acid Confers Neuroprotection in Rats With Acute Cervical SCI and Attenuates NLRP3/Caspase‐1/GSDMD Pyroptotic Signaling
Source: J Biochem Mol Toxicol. 2026 Oct 28;40(11):e71157. doi: 10.1002/jbt.71157 (PMC13717287; doi:10.1002/jbt.71157)
Supplement: Supplementary file 1 [file JBT-40-e71157-s001.docx]

# **Supplementary Material**

**Table 1 Effects of different concentrations of UDCA on BV2 cell viability (‾X±SD，n=3)**

| Concentrations of UDCA (μmol/L) | BV2 Cell Viability (%) |
| --- | --- |
| 0 | 99.85±5.29* |
| 12.5 | 98.73±1.71 |
| 25 | 98.73±4.66 |
| 50 | 103.60±1.1** |
| 100 | 91.28±1.94 |

Compared with 100 μmol/L, **P*＜0.05, ***P*＜0.01

**Table 2 Effects of Different Concentrations of UDCA Combined with LPS on the Viability of BV2 Cells (‾X±SD，n=3)**

| Concentrations of UDCA (μmol/L) | Cell viability of BV2 cells cultured with LPS (%) |
| --- | --- |
| 0 | 93.14±2.82 |
| 12.5 | 94.26±2.33 |
| 25 | 102.80±6.55 |
| 50 | 96.50±1.71 |
| 100 | 93.14±1.71 |

**Table 3 Average fluorescence intensity of NLRP3 in BV2 cells across each group (‾X±SD，n=4)**

| Group | Average fluorescence intensity |
| --- | --- |
| Control | 1.00±0.29**** |
| LPS | 4.40±0.09 |
| LPS+UDCA | 3.72±0.39* |

Compared with the LPS group, **P*＜0.05, *****P*＜0.0001

**Table 4 Percentage of Cavity Area in Spinal Cord Tissues of Each Group of Rats (‾X±SD，n=6)**

| group | Cavity Area (%) |
| --- | --- |
| Sham | 1.68±2.20**** |
| SCI | 13.74±4.03 |
| SCI+UDCA | 6.39±2.55** |

Compared with the SCI group，***P*＜0.01, *****P*＜0.0001

**Table 5 Statistics on the usage rate of the affected forelimb at different observation time points for each group of rats (‾X±SD，n=6)**

| Group | Time | | | | |
| --- | --- | --- | --- | --- | --- |
|  | Baseline (%) | postoperative days 1 (%) | postoperative days 3 (%) | postoperative days 5 (%) | postoperative days 7 (%) |
| Sham | 54.34±9.15 | 38.58±5.01  **** ^aaa bbbb^ | 45.93±6.02  **** ^bb^ | 53.61±7.39  **** ^###^ | 57.55±8.53  **** ^#### &&^ |
| SCI | 50.84±5.59 | 0.11±0.26  ^bb^ | 0.87±1.36  ^b^ | 3.47±4.93 | 12.03±4.10  ^## &^ |
| SCI+UDCA | 51.49±4.46 | 0.28±0.69  ^aaaa bbbb^ | 5.23±7.13  ^aa bbbb^ | 18.27±9.91  *** ^#### && bbb^ | 33.30±2.88  **** ^#### &&&& aaa^ |

Compared with the SCI group at the same postoperative time point, ***P* < 0.01, ****P* < 0.001, *****P* < 0.0001. Compared with the results obtained 1 day postoperatively within the same group, ^##^*P* < 0.01, ^###^*P* < 0.001, ^####^*P* < 0.0001.

Compared with the results obtained 3 day postoperatively within the same group, ^&^*P* < 0.05, ^&&^*P* < 0.01, ^&&&&^*P* < 0.0001.

Compared with the results obtained 5 day postoperatively within the same group, ^aa^*P* < 0.01, ^aaa^*P* < 0.001, ^aaaa^*P* < 0.0001.

Compared with the results obtained 7 day postoperatively within the same group, ^b^*P* < 0.05, ^bb^*P* < 0.01, ^bbb^*P* < 0.001, ^bbbb^*P* < 0.0001.

**Table 6 Relative expression of pyroptosis-related proteins in spinal cord tissues from each group of rats (‾X±SD，n=6)**

| Group | NLRP3 | Caspase-1 | GSDMD | N-GSDMD |
| --- | --- | --- | --- | --- |
| Sham | 0.51±0.27* | 0.76±0.28 | 1.00±0.13* | 0.88±0.17* |
| SCI | 0.94±0.28 | 1.12±0.23 | 1.33±0.26 | 1.31±0.21 |
| SCI+UDCA | 0.50±0.19* | 0.94±0.26 | 0.78±0.20*** | 1.11±0.29 |

Compared with the SCI group，**P*＜0.05, ****P*＜0.001

**Table** **7** **Relative fluorescence intensity of spinal cord Iba-1 (‾X±SD，n=6)**

| Group | Average fluorescence intensity |
| --- | --- |
| Sham | 1.00±0.22*** |
| SCI | 1.61±0.26 |
| SCI+UDCA | 1.28±0.08* |

Compared with the SCI group, **P*＜0.05, ****P*＜0.001

**Table 8 Serum IL-18, IL-1β and TNF-α levels in each group of rats (‾X±SD，n=5)**

| Group | IL-18 (pg/ml) | IL-1β (pg/ml) | TNF-α (pg/ml) |
| --- | --- | --- | --- |
| Sham | 74.58±1.58**** | 9.91±2.63* | 189.5±10.62* |
| SCI | 115.0±8.34 | 32.46±20.42 | 215.1±17.03 |
| SCI+UDCA | 78.11±9.52**** | 10.53±2.96* | 190.8±12.92* |

Compared with the SCI group，**P*＜0.05, *****P*＜0.0001
